# Elevated reticulocyte count – a clue to the diagnosis of haemolytic-uraemic syndrome (HUS) associated with gemcitabine therapy for metastatic duodenal papillary carcinoma: a case report

**DOI:** 10.1038/sj.bjc.6690242

**Published:** 1999-03

**Authors:** S Serke, H Riess, H Oettle, D Huhn

**Affiliations:** Department of Haematology–Oncology, Humboldt-Universität, Augustenburger Platz 1, Berlin, 13353, Germany

**Keywords:** gemcitabine, haemolytic-uraemic syndrome (HUS), pulmonary oedema

## Abstract

In adults, the haemolytic-uraemic syndrome (HUS) is associated with probable causative factors in the minority of all cases. Cytotoxic drugs are one of these potential causative agents. Although metastatic cancer by itself is a recognized risk-factor for the development of HUS, therapy with mitomycin-C, with *cis*-platinum, and with bleomycin carries a significant, albeit extremely small, risk for the development of HUS, compared with all other cytotoxic drugs. Gemcitabine is a novel cytotoxic drug with promising activity against pancreatic adenocarcinoma. We are reporting on one patient with metastatic duodenal papillary carcinoma developing HUS while on weekly gemcitabine therapy. The presenting features in this patient were non-cardiac pulmonary oedema, renal failure, thrombocytopenia and haemolytic anaemia. The diagnosis of HUS was made on the day of admission of the patient to this institution. Upon aggressive therapy, including one single haemodialysis and five plasmaphereses, the patient recovered uneventfully, with modestly elevated creatinine-values as a remnant of the acute illness. Re-exposure to gemcitabine 6 months after the episode of HUS instituted for progressive carcinoma, thus far has not caused another episode of HUS. © 1999 Cancer Research Campaign


*British Journal of Cancer* (1999) **79**(9/10), 1519–1521
© 1999 Cancer Research Campaign
Article no. bjoc.1998.0242

### Elevated reticulocyte count – a clue to the diagnosis of haemolytic-uraemic syndrome (HUS) associated with gemcitabine therapy for metastatic duodenal papillary carcinoma: a case report

**S Serke, H Riess, H Oettle and D Huhn**

Department of Haematology–Oncology, Charite, Campus Virchow-Klinikum, Humboldt-Universität, Augustenburger Platz 1, 13353 Berlin, Germany

**Summary** In adults, the haemolytic-uraemic syndrome (HUS) is associated with probable causative factors in the minority of all cases. Cytotoxic drugs are one of these potential causative agents. Although metastatic cancer by itself is a recognized risk-factor for the development of HUS, therapy with mitomycin-C, with *cis*-platinum, and with bleomycin carries a significant, albeit extremely small, risk for the development of HUS, compared with all other cytotoxic drugs. Gemcitabine is a novel cytotoxic drug with promising activity against pancreatic adenocarcinoma. We are reporting on one patient with metastatic duodenal papillary carcinoma developing HUS while on weekly gemcitabine therapy. The presenting features in this patient were non-cardiac pulmonary oedema, renal failure, thrombocytopenia and haemolytic anaemia. The diagnosis of HUS was made on the day of admission of the patient to this institution. Upon aggressive therapy, including one single haemodialysis and five plasmaphereses, the patient recovered uneventfully, with modestly elevated creatinine-values as a remnant of the acute illness. Re-exposure to gemcitabine 6 months after the episode of HUS instituted for progressive carcinoma, thus far has not caused another episode of HUS.

**Keywords:** gemcitabine; haemolytic-uraemic syndrome (HUS); pulmonary oedema

The haemolytic-uraemic syndrome (HUS) is the consequence of one single pathogenic process, leading to either thrombotic-thrombocytopenic purpura (TTP), or to microangiopathic haemolytic anemia (MAHA), or to adult HUS (Streiff and Bell, 1993). The aetiology remains unknown, but a number of predisposing conditions have been recognized. Cytotoxic drugs have been identified as playing a causative role in the development of HUS in cancer patients. Gemcitabine is a newly developed nucleoside analogue with high activity against pancreatic adenocarcinoma. The diagnosis of HUS was made in a patient treated with gemcitabine for metastatic papillary carcinoma for about 10 months. The initial clinical hint indicating the development of HUS in this patient, the sudden onset of anaemia and thrombocytopenia, was erroneously considered to be the result of myelotoxicity exerted by gemcitabine. The strongly elevated reticulocyte count alone, however, enabled a diagnosis of a hyperregenerative anaemia due to haemolysis, ruling out myelotoxicity as the cause of the cytopenia.

### CASE REPORT

In December 1994, the clinical diagnosis of a pancreatic carcinoma of the head of the pancreas was made in the 40-year-old male patient. He was operated on (Whipple-procedure), and the final diagnosis was that of a papillary carcinoma. No further therapy was administered. In August 1996, the patient was seen at an out-patient department, as an increase in CA 19-9 had been observed since June 1996. The first recorded elevated value of CA 19-9 was 5440 units $l^{-1}$ (normal upper limit = 25 U $l^{-1}$). Abdominal ultrasound evidenced two intrahepatic lesions (about 2 cm in diameter) not detected on an examination performed in May 1996. The diagnosis of metastatic papillary carcinoma was made, and the patient was offered an experimental therapy with gemcitabine. Treatment was given as typical weekly infusions over 30 min, with a dose of 1600 mg $day^{-1}$ for 3 weeks, followed by 1 week without gemcitabine. The drug was well tolerated, and no toxicity was observed. In January 1997, the hepatic lesions had decreased to about 0.5 cm in diameter (by ultrasound), and CA 19-9 was within the normal range. In June 1997, with a total dose of 53 400 mg of gemcitabine, a first episode of thrombocytopenia, 72 000 $\mu l^{-1}$, was observed. Until this date, the platelet count had been between 175 000 $\mu l^{-1}$ and 685 000 $\mu l^{-1}$, with a median of 478 000 $\mu l^{-1}$. At the same time, since August 1996 the LDH had increased from normal values to 507 U $l^{-1}$ (upper normal limit of 240 U $l^{-1}$). The creatinine, below 1.0 mg $dl^{-1}$ until this date, increased to 1.3 mg $dl^{-1}$. The haemoglobin value, ranging from 10.5 g $dl^{-1}$ to 14.7 g $dl^{-1}$ since August 1996, decreased to 8.2 g $dl^{-1}$. Notably, at this time no evidence of progressive disease was present. Attributing the thrombocytopenia and the anaemia to myelotoxicity of gemcitabine, the therapy was discontinued. At the end of August 1997, with gemcitabine discontinued for 7 weeks, the laboratory values had worsened: Plt 101 000 $\mu l^{-1}$; Hb 7.2 g $dl^{-1}$, creatinine 2.9 mg $dl^{-1}$, LDH 1190 U $l^{-1}$, urea 84 mg $dl^{-1}$, bilirubin 1.7 mg $dl^{-1}$. On the day of admission to this hospital, the laboratory values were: urea 124 mg $dl^{-1}$, creatinine 4.1 mg $dl^{-1}$, bilirubin 1.3 mg $dl^{-1}$, LDH 1440 U $l^{-1}$, plt 100 000 $\mu l^{-1}$, Hb 5.6 g $dl^{-1}$, schistocytes 2.7% of all RBC, reticulocytes 18% of all RBC. All coagulation parameters

*Received 29 June 1998*
*Revised 26 September 1998*
*Accepted 9 October 1998*

*Correspondence to:* S Serke were within the normal range. Examination of the urine disclosed no abnormality other than a slight proteinuria (500 mg per 1 l). The patient was orthopnoic, but auscultation of the lungs disclosed no abnormality with the exception of bilateral symmetric basal pleural effusion; the heart sounds were weak, and by ultrasound a circular pericardiac effusion of about 0.9 cm was detected. Bilateral ankle oedema (having developed within the last 2 weeks prior to admission) were evident, and the arterial blood pressure was 210/115 mmHg as measured on both arms.

Having made the clinical diagnosis of adult HUS in a cancer patient treated with gemcitabine, therapy was initiated with 2 units of fresh frozen plasma and 2 units of packed RBC, combined with the administration of nifedipine and furosemide. As the creatinine value increased to 5.8 mg $dl^{-1}$ within 12 h and the orthopnoea worsened, one single haemodialysis was performed after 12 h from admission, causing a weight loss of 4.0 kg. After the dialysis, the first plasmapheresis was performed, with substitution of 4 l of fresh frozen plasma. Following this plasmapheresis, all laboratory values improved, and the dyspnoea decreased gradually. Additional plasmaphereses with substitution of fresh frozen plasma were performed likewise on days 2, 4, 5 and 6. The arterial blood pressure normalized within 10 days and nifedipine was discontinued. Thereafter, plasmaphereses were performed twice per week until the demission of the patient on day 24. At demission, creatinine was 2.4 mg $dl^{-1}$, urea 69 mg $dl^{-1}$, LDH 280 U $l^{-1}$, Hb 12.8 g $dl^{-1}$, and all other laboratory values had normalized, including reticulocytes at 2.0% of all RBC and plt of 229 000 $\mu l^{-1}$.

From demission on 4 October 1997, the patient was doing well, but laboratory abnormalities persisted: creatinine 1.8 mg $dl^{-1}$, urea 49 mg $dl^{-1}$ (upper normal limit 45 mg $dl^{-1}$), LDH 285 U $l^{-1}$, Hb 12.4 g $dl^{-1}$ (values from 11 November 1997). Early in 1998, CA 19-9 gradually increased to 1965 U $l^{-1}$, and the hepatic lesions again gained in size. The patient was given 5-fluorouracil, and severe diarrhoea developed. After recovery from the diarrhoea, the patient has again been given gemcitabine. Currently, with another 3000 mg of gemcitabine administered, the patient is doing well without any signs of recurrence of HUS.

### DISCUSSION

It was reported as early as 1962 that HUS occurs associated with widespread metastatic cancer in patients not receiving chemotherapy (Brain et al, 1962). In 1980, the first report of a clinical condition best referred to as HUS in patients treated with mytomycin-C was published (Gulati et al, 1980). Since this report, a growing number of chemotherapeutic agents, among those *cis*-platinum and bleomycin, have been linked to the development of HUS (Nordstrom and Strang, 1993). The etiology of HUS is unknown, but endothelial damage may lead to extensive formation of microthrombi in smaller vessels (Matsumae et al, 1996; Groff et al, 1997). Von Willebrand factor multimers of supranormal size, a factor predisposing to thrombosis, have been described in patients with TTP and with HUS (Furlan, 1996; Zeigler et al, 1996). Although proof is lacking, the thrombocytopenia and the haemolysis are attributed to simple mechanical destruction of these cells in the microcirculation. The well-vascularized kidney appears to be the major parenchymatic target for this process of formation of microthrombi, causing acute renal failure and arterial hypertension of sudden onset. One feature distinguishing adult HUS from TTP and from childhood HUS is the development of non-cardiogenic pulmonary oedema, and focal fibrosing alveolitis with alveolar septal oedema, capillary congestion and fibrin deposition have been described (Crocker and Jones, 1983).

The diagnosis of adult HUS is made on the base of the combination of hyperregenerative thrombocytopenia, haemolytic anaemia, renal failure with arterial hypertension of sudden onset, and pulmonary oedema. Neurologic complications, a hallmark for the diagnosis of TTP, are infrequent.

Therapy of this potentially fatal condition, with a mortality of 25% despite adequate treatment, as reported in one series of 68 patients treated at one single institution (Conlon et al, 1995), and with a mortality of 37% in another single-institution series reporting on 35 patients (Sens et al, 1997), remains controversial, but plasma infusions and/or plasmapheresis are now recognized as the modalities with major potential benefit (Sens et al, 1997; DeWit et al, 1996; Keller et al, 1994).

Gemcitabine has a structure similar to that of cytosine-arabinoside, a drug not associated with HUS, and this drug has a well-documented efficacy against adenocarcinoma of the pancreas, with minor efficacy against other types of cancer. In one recent review article (Green, 1996) evaluating toxicity in 790 patients treated with single-agent gemcitabine, HUS is not mentioned, but ‘a few cases of renal failure of uncertain etiology’ are reported. Interestingly, although again HUS has not been reported by these authors, in a second large series of patients participating in phase II studies (Tonato et al, 1995) it is reported that peripheral oedema not explainable by renal, hepatic or cardiac dysfunction is observed in about 10% of patients. In a further series of 360 patients, it is likewise emphasized that the development of peripheral oedema was never associated with cardiac, hepatic or renal failure (Cortes-Funes et al, 1997). It is tempting to speculate that these oedema might be due to disturbances in the microcirculation, caused by gemcitabine. In this regard the report of Dobbie et al on the development of veno-occlusive disease in a patient on gemcitabine appears to be of particular interest (Dobbie et al, 1998).

In one series of 82 patients on single-agent gemcitabine, it has been reported that two patients developed acute renal failure 4 and 6 weeks after the last dose of gemcitabine (Anderson et al, 1994). We were unable to trace any publications reporting on cases of HUS associated with gemcitabine therapy other than those of Casper et al (1994) and of Brodowicz et al (1997). Giuseppe Giaccone has reported on the occasion of the 9th European Congress for Clinical Oncology (Hamburg, Germany, September 1997) that among 60 patients with advanced NSC-LC treated in a phase I/II trial with paclitaxel and gemcitabine, one patient developed haemolytic anaemia, and one patient died owing to thrombotic-thrombocytopenic purpura (minutes from a satellite symposium sponsored by Bristol-Myers Squibb Company; host: Siegfried Seeber). The company marketing gemcitabine states in an extended data sheet of its product (GEMZAR®) that ‘clinical findings consistent with HUS were reported in 6 of 2429 patients (0.25%)’. (Eli Lilly, 1997), probably referring to data-on-file of patients participating in clinical trials sponsored by this company.

In view of the ill-defined aetio-pathological events leading to the development of adult HUS in cancer patients, there is no proof at all that the HUS diagnosed in the patient described by us can be attributed solely to the therapy with gemcitabine. One alternative cause for the induction of HUS, the metastatic papillary adenocarcinoma itself, is not very probable, as ultrasound examination disclosed regression of the previously described hepatic lesions

 *© Cancer Research Campaign 1999*

and normalization of the strikingly elevated CA 19-9 values until admission of this patient for HUS. Another alternative cause may be other drugs: the patient was on propanolol medication for 4 years (continued until today) and prior to each infusion of gemcitabine he had been given alizaprid (VERGENTAN®). We are unable, however, to trace any publications on HUS and either of these two drugs. Thus, gemcitabine is the most probable cause of HUS in this patient.

The patient described by us had been on gemcitabine for almost 12 months, and a cumulative dose of 53 400 mg had been administered. A dose–response relationship is well documented in the setting of mitomycin-C-induced HUS (Verwey et al, 1987).

Rapid diagnosis of this rare disorder and rapid institution of adequate therapy might be the reason for the satisfactory outcome of the episode of HUS in the patient described by us. There is little doubt, however, that discontinuation of the drug is also mandatory. We would like to emphasize, however, that in our patient it took about 8 weeks from the first laboratory hints suggesting HUS (in retrospect) to the development of the full-blown clinical condition. During these 8 weeks, the patient had not been treated with gemcitabine.

We are suggesting, however, despite the low incidence of HUS associated with gemcitabine, inclusion of additional prescribing information for this drug. Hyperregenerative haemolytic anaemia is one hallmark of HUS, and reticulocytes, therefore, are generally elevated in HUS. Thus, in a patient on gemcitabine therapy who developes anaemia, a reticulocyte count seems mandatory. In our patient, the first episode of thrombocytopenia and anaemia was attributed to myelotoxicity of gemcitabine. This myelotoxicity is much more frequent than HUS (anaemia: grade I and II in 55%, grade III and IV in 10%; thrombocytopenia: grade I and II in 37%, grade III and IV in 10%) (GEMZAR prescribing information, Eli Lilly). The simple and reliable test for reticulocytes is capable of differentiating between the hyporegenerative anaemia due to the myelotoxicity of gemcitabine and the hyperregenerative haemolytic anaemia due to HUS associated with gemcitabine. Although a corresponding test is available for 'reticulated platelets' (Kienast and Schmitz, 1990), this test cannot be recommended as a routine clinical-laboratory test. We therefore recommend performing reticulocyte-counting as part of the safety assessment in each patient on gemcitabine therapy who develops anaemia or thrombocytopenia.

### REFERENCES

Anderson H, Lund B, Bach F, Thatcher N, Walling J and Hansen HH (1994) Single-agent activity of weekly gemcitabine in advanced non-small-cell lung cancer: a phase II study. *J Clin Oncol* **12**: 1821–1826

Brain MC, Dacie JV and Hourihane OB (1962) Microangiopathic hemolytic anemia: the possible role of vascular lesions in pathogenesis. *Br J Haematol* **8**: 358–364

Brodowicz T, Breiteneder S, Witschke C and Zielinski CC (1997) Gemcitabine-induced hemolytic uremic syndrome: a case report (letter). *J Natl Cancer Inst* **89**: 1895–1896

Casper ES, Green MR, Kelsen DP, Heelan RT, Brown TD, Flombaum CD, Trochanowski B and Tarassoff PG (1994) Phase II trial of gemcitabine (2,2′-difluorodeoxycytidine) in patients with adenocarcinoma of the pancreas. *Invest New Drugs* **12**: 29–34

Conlon PJ, Howell DN, Macik G, Kovalik EC and Smith SR (1995) The renal manifestations and outcome of thrombotic thrombocytopenic purpura/hemolytic uremic syndrome in adults. *Nephrol Dial Transplant* **10**: 1189–1193

Cortes-Funes H, Martin C, Abratt R and Lund B (1997) Safety profile of gemcitabine, a novel anticancer agent, in non-small-cell lung cancer. *Anticancer Drugs* **8**: 582–587

Crocker J and Jones EL (1983) Haemolytic-uraemic syndrome complicating long-term mitomycin C and 5-fluorouracil therapy for gastric carcinoma. *J Clin Pathol* **36**: 24

DeWit M, Weh HJ and Hossfeld DK (1996) Thrombotic thrombocytopenic purpura and hemolytic uremic syndrome. *Med Welt* **47**: 528–532

Dobbie M, Hofer S, Oberholzer M and Herrmann R (1998) Veno-occlusive disease of the liver induced by gemcitabine (letter). *Ann Oncol* **9**: 681

Furlan M (1996) Von Willebrand factor: molecular size and functional activity. *Ann Hematol* **72**: 341–348

Green MR (1996) Gemicitabine safety overview. *Semin Oncol* **23**(Suppl 10): 32–35

Groff JA, Kozak M, Boehmer JP, Demko TM and Diamond JR (1997) Endotheliopathy: a continuum of hemolytic uremic syndrome due to mitomycin therapy. *Am J Kidney Dis* **29**: 280–284

Gulati SC, Sordillo P, Kempin S et al (1980) Microangiopathic hemolytic anemia observed after treatment of epidermoid carcinoma with mitomycin C and 5-fluorouracil. *Cancer* **45**: 2252

Keller F, Schwarze H and Schwarz A (1994) Clinical management of hemolytic-uremic syndrome and thrombotic-thrombocytopenic purpura. *Wien Klin Wochenschr* **106**: 603–607

Kienast J and Schmitz G (1990) Flow cytometric analysis of thiazole orange uptake by platelets: a diagnostic aid in the evaluation of thrombocytopenic disorders. *Blood* **75**: 116–121

Matsumae T, Takebayashi S and Naito S (1996) The clinico-pathological characteristics and outcome in hemolytic-uremic syndrome of adults. *Clin Nephrol* **45**: 153–162

Nordstrom B and Strang P (1993) Microangiopathic hemolytic anemias (MAHA) in cancer. A case report and review. *Anticancer Res* **13**: 1845–1850

Sens YAS, Miorin LA, Silva HGC, Malheiros DMAC, Filho DM and Jabur P (1997) Acute renal failure due to hemolytic-uremic syndrome in adult patients. *Renal Fail* **19**: 279–282

Streiff M and Bell WR (1993) Thrombotic thrombocytopenic purpura – hemolytic uremic syndrome. *Curr Opin Hematol* **1**: 274

Tonato M, Mosconi AM and Martin C (1995) Safety profile of gemcitabine. *Anticancer Drugs* **6** (Suppl. 6): 27–32

Verwey J, De Vries J and Pinedo HM (1987) Mitomycin C-induced renal toxicity, a dose-dependent side effect? *Eur J Cancer* **23**: 195

Zeigler ZR, Rosenfeld CS, Andrews III DF, Nemunaitis J, Raymond JM, Shadduck RK, Kramer RE, Gryn JF, Rintels PB, Besa EC and George JN (1996) Plasma von Willebrand factor antigen (vWF:AG) and thrombomodulin (TM) levels in adult thrombotic thrombocytopenic purpura/hemolytic uremic syndromes (TTP/HUS) and bone marrow transplant-associated microangiopathy (BMT-TM). *Am J Hematol* **53**: 213–220

© Cancer Research Campaign 1999
